# Supplementary material for: Multi-omics approach identifies germline regulatory variants associated with hematopoietic malignancies in retriever dog breeds
Source: PLoS Genet. 2021 May 13;17(5):e1009543. doi: 10.1371/journal.pgen.1009543 (PMC8118335; doi:10.1371/journal.pgen.1009543)
Supplement: S1 Fig — (PDF) [file pgen.1009543.s002.pdf]

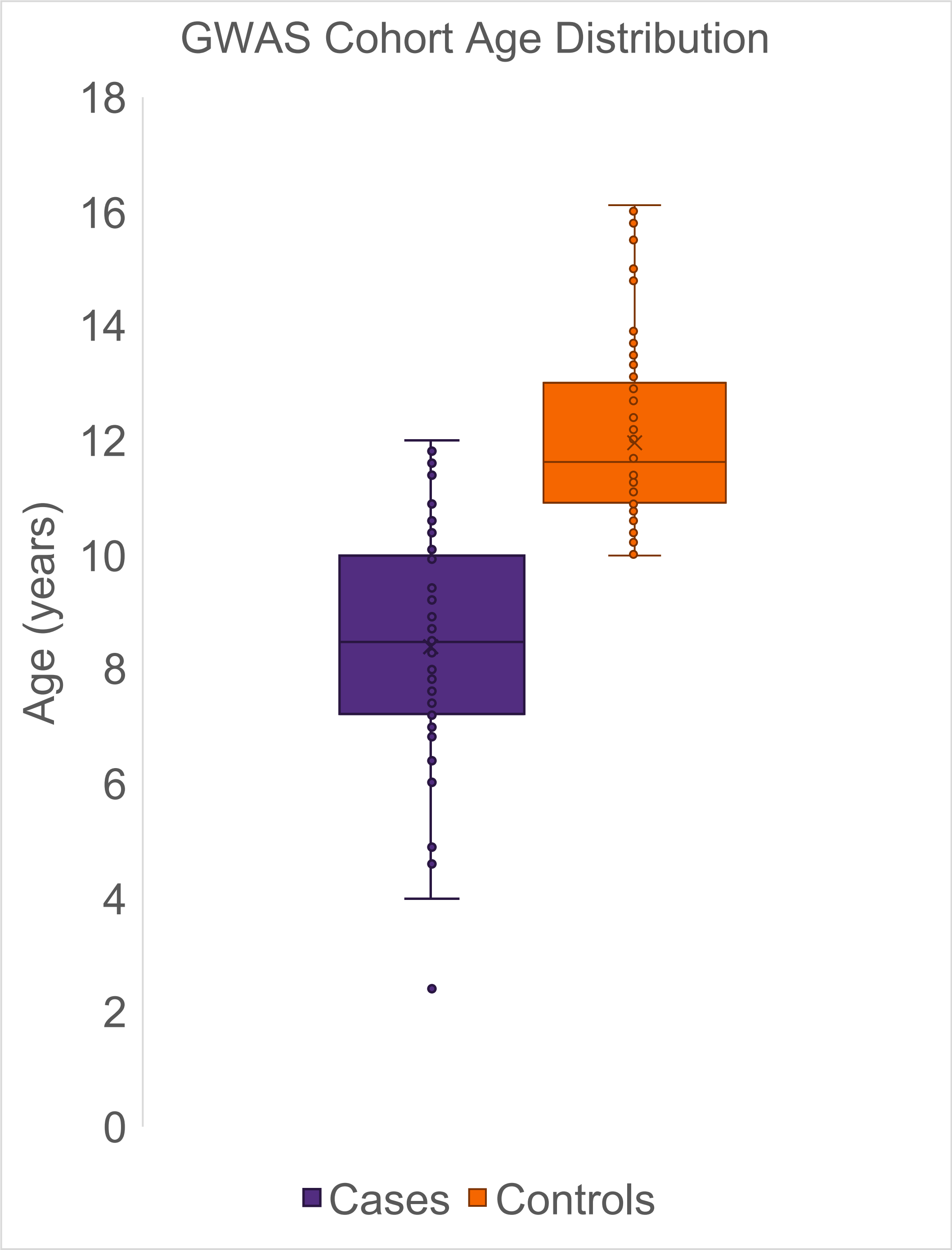

Fig S1. Age of FCRs in the GWAS cohort. Box plots are shown for case (n=68) age at diagnosis (left) and control (n=132) age at collection (right) with median ages of 8.5 and 11.6 years, respectively.
