# Supplementary material for: Multi-omics approach identifies germline regulatory variants associated with hematopoietic malignancies in retriever dog breeds
Source: PLoS Genet. 2021 May 13;17(5):e1009543. doi: 10.1371/journal.pgen.1009543 (PMC8118335; doi:10.1371/journal.pgen.1009543)
Supplement: S2 Fig — (PDF) [file pgen.1009543.s003.pdf]

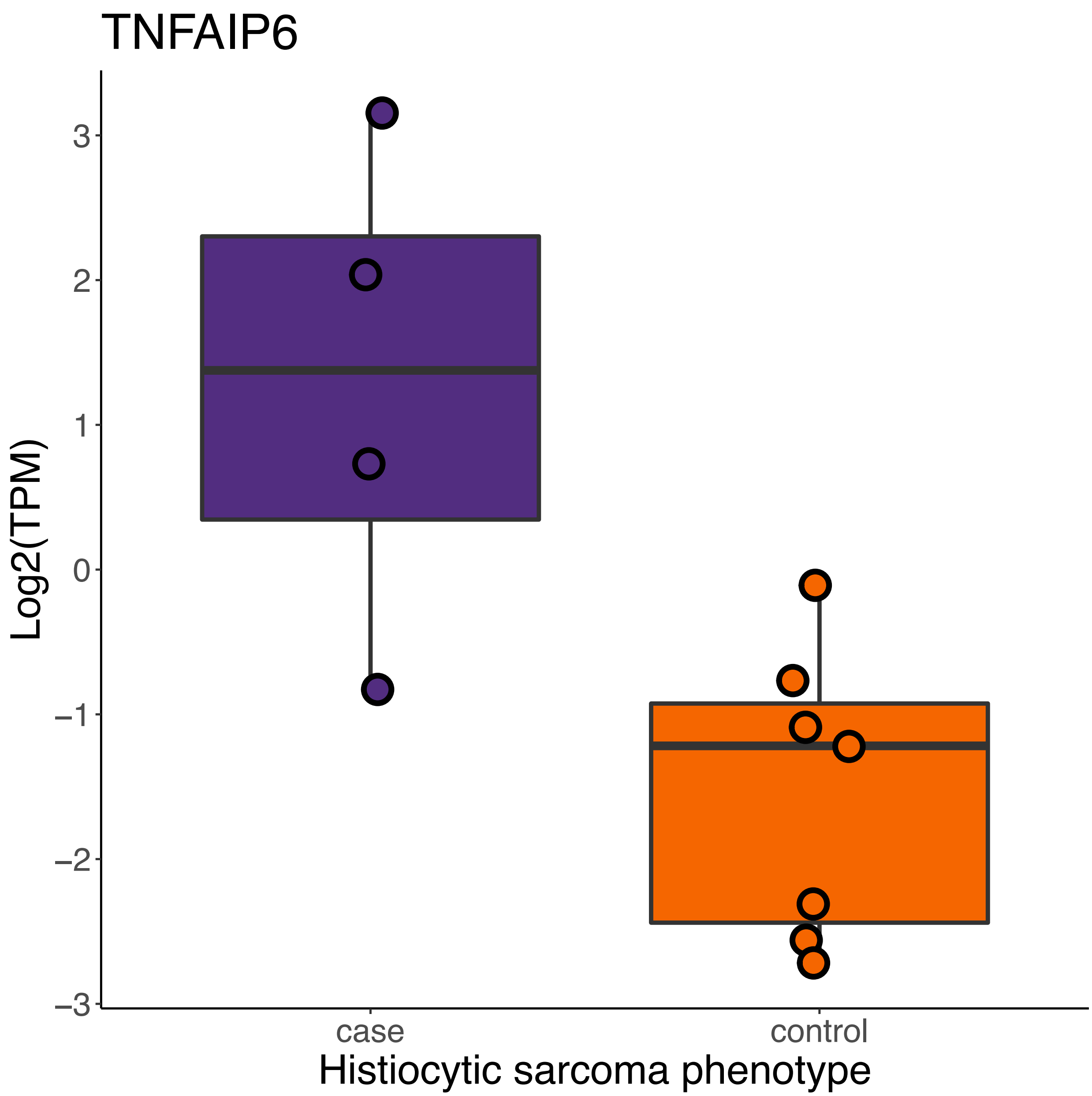

Fig S2. Boxplots of transcripts per million counts for *TNFAIP6*. Log2(transcripts per million) values are plotted on the y-axis for the four FCR cases homozygous for CFA19 risk (purple) and all seven FCR controls (orange), Wilcoxon *P*-value=0.024.
